# Xylanase and stimbiotic supplementation improve broilers performance and nutrient digestibility across both wheat-barley and corn-based diets

**DOI:** 10.1016/j.psj.2025.105224

**Published:** 2025-04-29

**Authors:** Mehdi Toghyani, Eunjoo Kim, Shemil P. Macelline, Gemma González-Ortiz, Reza Barekatain, Sonia Y. Liu

**Affiliations:** a School of Life and Environmental Sciences, Faculty of Science, The University of Sydney, Camperdown 2006 NSW, Australia; b Poultry Research Foundation, The University of Sydney, Camden 2570 NSW, Australia; c AB Vista, Marlborough, Wiltshire, SN8 4AN, UK; d South Australian Research and Development Institute, Roseworthy Campus, Roseworthy, SA, Australia

**Keywords:** Grain source, Xylanase, Stimbiotic, Viscosity, Intestinal permeability

## Abstract

The present study investigated the effects of supplemental xylanase or stimbiotic in male broiler chickens fed either corn- or wheat-barley-based diets. A total of 1,296 Ross 308 day-old chicks were assigned to a 2 × 3 factorial design, evaluating the effects of diet grain source (wheat-barley or corn) and additives (none, xylanase, or stimbiotic). The stimbiotic used in the present study contained xylanase and fermentable xylo-oligosaccharides. Each treatment was replicated 8 times, with 27 birds per replicate pen. At day 21, 3 birds per pen were selected for blood sample collection and another 3 birds at day 27 for digesta collection. The final body weight at day 42 was not statistically affected by grain source, additive supplementation, or their interaction (*P* > 0.05). Over the entire production period (0–42 d), an interaction between grain source and additive supplementation was found for feed conversion ratio (FCR), where xylanase or stimbiotic improved the FCR across the diet type, with a more pronounced improvement achieved when supplemented to the wheat-barley based diets (*P* < 0.01). A feed grain × additive interaction resulted in lower total feed intake in birds fed the wheat-barley based diets only in response to stimbiotic (*P* < 0.05). Ileal viscosity was also affected by an interaction between grain source and additive supplementation, in which viscosity reduction by xylanase or stimbiotic was only achieved in birds fed the wheat-barley based diets (*P* < 0.01). Similarly, an interaction was found in the ileal digestibility coefficient of protein (*P* = 0.016) and starch (*P* = 0.006), where either xylanase or stimbiotic improved the digestibility only in birds offered the wheat-barley based diets. Serum fluorescein isothiocyanate dextran level was higher in birds fed the corn-based diets compared to those fed the wheat-barley based diets (*P* < 0.01). These results suggest that dietary supplementation with either xylanase or stimbiotic improved feed efficiency, regardless of the dietary grain source, likely through enhanced nutrient digestibility and/or reduced digesta viscosity.

## Introduction

Feed grain cell walls are primarily composed of fibre, referred to as non-starch polysaccharides (**NSP**). Accordingly, significant amounts of NSP are inherently present in plant-based poultry diets; however, they are indigestible by chickens' endogenous enzymes. As a result, chickens largely rely on microbial fermentation for fibre digestion. Hence, certain types of NSP can negatively impact feed efficiency. NSP in soluble forms can increase small intestinal digesta viscosity, which hinders the diffusion of endogenous enzymes and depresses nutrient utilisation (Nguyen et al., 2022). Furthermore, the complex structure of NSP can trap starch and amino acids within their matrix, decreasing their bioavailability for birds (Choct, 2015). Nutritional strategies are thus required to minimize these anti-nutritive impacts of NSP on poultry performance.

One such strategy is supplementing fibre-degrading enzymes to plant-based poultry diets (Bedford et al., 2022). Fibre-degrading enzymes can break down and/or loosen NSP structure so that they can be better utilised by intestinal bacteria. For instance, xylanase is an enzyme that randomly cleaves the backbone of long xylan chains, releasing soluble xylans and in some cases xylo-oligosaccharides (Morgan et al., 2020). Then, these xylo-oligosaccharides can be further fermented by gut microbiota, producing volatile fatty acids (Nguyen et al., 2021). This bioconversion exerts prebiotic effects in birds by selectively fuelling beneficial gut bacteria with fermentable substrates (Kim et al., 2022; Nieto-Domínguez et al., 2017). Consequently, a novel approach has emerged, involving the combined use of NSP-degrading enzymes and fermentable oligosaccharides to maximize the prebiotic effects. However, stimbiotics specifically signal changes in fermentative activity at concentrations far too low to be of significant value as a substrate *per se* (González-Ortiz et al., 2019; Davies et al., 2024). Hence, stimbiotic supplementation would be expected to protect the gut more efficiently from pathogenic bacterial colonization compared to supplementing just xylanase alone by changing the gut fermentative capacity.

Corn and wheat are the primary cereal grains used in broiler diets as energy sources due to their high starch content. However, these grains differ significantly in their NSP content and composition. Wheat typically contains a higher total NSP content, including both soluble and insoluble fractions, compared to corn. The NSP profile of wheat is dominated by arabinoxylans, whereas corn contains more cellulose and lower levels of soluble arabinoxylans (Annison, 1991). Exogenous xylanase has historically been added to wheat-based diets for broiler chickens to enhance growth performance by reducing digesta viscosity through the depolymerization of soluble NSPs, which improves nutrient digestibility and metabolizable energy utilization (Bedford et al., 2022; Gonzalez-Ortiz et al., 2017). Interestingly, xylanase supplementation has also been shown to improve growth performance, particularly feed efficiency, in broilers fed corn-based diets, despite having minimal impact on intestinal viscosity or nutrient digestibility (Amerah et al., 2017; Kiarie et al., 2014). The effectiveness of xylanase in corn-based diets is attributed to its ability to degrade both insoluble and soluble arabinoxylans in corn cell walls, releasing arabinoxylo-oligosaccharides (AXOS). These AXOS act as prebiotics, promoting the proliferation of beneficial gut microbiota such as *Lactobacillus* and *Bifidobacterium*, which support gut health and overall performance (Bedford and Cowieson, 2012).

This study hypothesized that combining xylanase and xylo-oligosaccharides as a stimbiotic, through a small dose of fermentable oligosaccharides, could enhance xylanase efficacy and thereby improve nutrient utilization in plant-based diets for broiler chickens. Therefore, the objective of this feeding trail was to examine the effects of xylanase or stimbiotic supplementation on growth performance, digesta characteristics, nutrient digestibility, and intestinal permeability in broilers fed corn- or wheat-barley-based diets.

## Materials and methods

### Experimental design, housing and diets

All experimental procedures were approved by the Animal Ethics Committee of the University of Sydney (Project number: 2021/2010).

Ross 308 off-sex male broiler chickens (*n* = 1296) were obtained from a commercial hatchery (Aviagen, Goulburn NSW, Australia). Upon arrival, all birds were weighed and allocated to 48 floor pens (2.25 m^2^) with fresh softwood pine shavings as a bedding material. Feed and water were available *ad libitum* on bell feeders and nipple drinkers. The experimental design was completely randomized with a 2 × 3 factorial arrangement of the treatments, with factors of two sources of grains (corn vs. wheat-barley) and three sources of additives (none, xylanase or stimbiotic). There were eight replicate pens per treatment, with 27 birds per replicate. There was no statistical difference in initial pen body weights across the treatments. The basal diets were based either on corn and soybean meal or wheat, barley and soybean meal, according to the nutrient specification for Ross 308 broilers (Aviagen, 2019; Table 1). Prior to diet formulation, representative subsamples of corn, wheat, barley, soybean meal, and canola meal were analyzed by near-infrared spectroscopy to predict proximate analysis, digestible amino acid concentrations, and metabolizable energy (ME) using AMINONIR®PROX, AMINONIR®NIR, and AMINONIR® NRG (Evonik Nutrition & Care, Rodenbacher Chaussee, Hanau-Wolfgang, Germany), respectively.

**Table 1:** The ingredients composition, calculated and analyzed nutrient specifications of experimental diets.

Ingredients (%)	Starter (0–14 d)		Grower (14–28 d)		Finisher (28–42 d)	
	Corn	Wheat	Corn	Wheat	Corn	Wheat
Corn, 8.5 %	58.5	–	59.4	–	66.2	–
Wheat, 10.0 %	–	53.3	–	44.7	–	40.4
Barley, 9.0 %	–	7.50	–	15.00	–	25.0
Soybean meal, 46.5 %	32.9	29.8	28.7	26.9	21.0	19.50
Canola meal, 37.5 %	4.00	4.00	6.00	6.00	7.00	7.00
Canola oil	1.40	2.25	2.95	4.60	3.10	5.65
Limestone 38 %	1.36	1.39	1.25	1.26	1.13	1.14
Mono Di-Ca Phosphate	0.600	0.470	0.480	0.340	0.360	0.200
Salt	0.270	0.225	0.215	0.180	0.180	0.165
DL-Methionine	0.250	0.235	0.235	0.215	0.210	0.195
Vit/Min premix^1^	0.200	0.200	0.200	0.200	0.200	0.200
L-Lysine HCl	0.175	0.235	0.205	0.230	0.225	0.245
Na bicarbonate	0.145	0.190	0.185	0.195	0.200	0.170
Choline chloride	0.080	0.080	0.070	0.070	0.060	0.060
L-Threonine	0.035	0.065	0.045	0.060	0.060	0.080
Phytase	0.010	0.010	0.010	0.010	0.010	0.010
TiO2	–	–	0.500	0.500	–	–
Calculated nutrient values (%, unless specified)						
AME, kcal/kg	2950	2950	3050	3050	3125	3125
NE, kcal/kg^2^	2327	2323	2424	2424	2497	2503
Crude protein	22.0	22.3	20.9	21.5	18.3	19.0
Digestible lysine	1.180	1.180	1.128	1.128	0.978	0.978
Digestible methionine	0.553	0.529	0.527	0.503	0.475	0.452
Digestible met + cys	0.861	0.861	0.824	0.824	0.743	0.743
Digestible threonine	0.743	0.743	0.711	0.711	0.636	0.636
Digestible isoleucine	0.836	0.837	0.780	0.792	0.662	0.673
Digestible tryptophan	0.236	0.257	0.220	0.245	0.183	0.209
Digestible arginine	1.334	1.324	1.245	1.259	1.047	1.064
Digestible valine	0.920	0.909	0.869	0.869	0.756	0.753
Phytate P	0.271	0.296	0.275	0.299	0.270	0.295
Ca	0.900	0.900	0.840	0.840	0.760	0.760
Available P	0.450	0.450	0.420	0.420	0.380	0.380
Total P	0.56	0.56	0.53	0.54	0.48	0.50
Na	0.200	0.200	0.190	0.190	0.180	0.180
Cl	0.250	0.250	0.220	0.220	0.200	0.210
K	0.824	0.816	0.760	0.772	0.648	0.669
DEB, mEq/kg	227.1	225.1	215.1	218.1	187.7	190.2
Analyzed values (%, unless specified)						
Gross Energy, kcal/kg	3945	4019	4062	4113	4115	4140
Crude protein	21.6	23.4	20.1	22.2	18.5	19.0
Ether extract	4.51	4.32	5.81	6.50	6.31	7.32
Starch	38.7	38.8	41.4	37.9	42.2	39.5
Total phosphorus	0.61	0.63	0.57	0.59	0.51	0.55

1 Vitamin concentrate supplied per kilogram of diet: retinol, 12000 IU; cholecalciferol, 5000 IU; tocopheryl acetate, 75 mg, menadione, 3 mg; thiamine, 3 mg; riboflavin, 8 mg; niacin, 55 mg; pantothenate, 13 mg; pyridoxine, 5 mg; folate, 2 mg; cyanocobalamin, 16 *μ*g; biotin, 200 *μ*g; cereal-based carrier, 149 mg; mineral oil, 2.5 mg. Trace mineral concentrate supplied per kilogram of diet: Cu (sulphate), 16 mg; Fe (sulphate), 40 mg; I (iodide), 1.25 mg; Se (selenate), 0.3 mg; Mn (sulphate and oxide), 120 mg; Zn (sulphate and oxide), 100 mg; cereal-based carrier, 128 mg; mineral oil, 3.75 mg.

2 Net energy; calculated NE = 0.808 *×* AMEn (MJ/kg) – 0.017 *×* crude protein (%) + 0.031 *×* ether extract

The xylanase treatment contained 16,000 BXU/kg (Econase XT 25, AB Vista, Marlborough, UK). The stimbiotic treatment contained the same activity of xylanase plus fermentable xylo-oligosaccharide (Signis, AB Vista, Marlborough, UK). Both additives were added on top of the basal diet at the rate of 100 g/t. All diets contained phytase (Quantum Blue 5G, AB Vista, Marlborough, UK) at 500 FTU/kg feed, and the matrix values for calcium, sodium and available phosphorus were applied. Diets were steam pelleted at 85 °C (ø = 2.5 mm), and starter diets were further crumbled. Starter diets were offered from 0 to 14 d, grower diets from 14 to 28 d, and finisher diets from 28 to 42 d.

### Data and sample collection

Pen body weights were recorded on 14, 28 and 42 days of age, feed intake was measured at similar intervals and used to calculate feed conversion ratio (FCR) corrected for mortality.

At day 21 of trial, three birds per pen close to the mean body weight of the pen were selected and orally gavaged with fluorescein isothiocyanate-dextran (FITC-d; 4.16 mg/kg live body weight). After 210 min, blood samples were collected from the birds treated with FITC-d into vacutainers containing lithium heparin for serum separation. At day 27, three birds per pen representing the mean body weight of the pen were euthanised by intravenous injections of sodium pentobarbitone to collect duodenal, jejunal and ileal contents for viscosity analysis, also ileal digesta samples were collected and pooled per replicate pen for nutrient digestibility analysis.

### Chemical analysis and calculations

Blood samples were centrifuged at 3000 × g at 4 °C for 10 min to separate the serum from the blood cells. Serum FITC-d levels were measured at the excitation wavelength of 485 nm and emission wavelength of 528 nm using a spectrophotometer, according to Barekatain et al. (2019). The fluorescence levels were then calculated using an equation from a standard curve with known FITC-d concentrations, corrected for values from FITC-d free birds as a blank. Diets and lyophilised digesta samples were ground to pass through a 0.5 mm sieve and analysed for dry matter, crude protein, gross energy, starch and titanium dioxide (TiO_2_). Dry matter contents were measured according to the standard method of AOAC (2012) (method 930.15). Nitrogen content was determined by the combustion method using a LECO TruMac® N analyzer (Leco Corporation, St. Joseph, MI), and the measured values were multiplied by a conversion factor of 6.25 to obtain crude protein values. The gross energy of diets and digesta were determined by bomb calorimetry using an adiabatic calorimeter (Parr 1281 bomb calorimeter, Parr Instruments Co., Moline, IL). Starch concentrations were analysed using total starch assay kits (Megazyme, Bray Business Park, Bray, Co. Wicklow, Ireland). The concentration of TiO_2_ was quantified according to the method described by Short et al. (1996) using a spectrophotometer at 410 nm (Cary 50 Bio UV-Visible spectrophotometer, Varian INC., Palo Alto, CA).

To determine digesta viscosity, the individual digesta samples from the duodenum, jejunum and ileum were centrifuged at 12,000 × g for 10 min at 4 °C, and about 0.5 mL of supernatant was used to measure viscosity with a Brookfield DVIII viscometer (AMETEK Brookfield, Middleboro, MA) at 25 °C with a CP 40 cone. The shear rate was from 5 to 500 s^−1^, over which the samples did not exhibit shear thinning.

Apparent ileal digestibility coefficient of nutrients was calculated using the following equation:

Digestibility coefficient = ((Nutrient/TiO_2_)_diet_-(Nutrient/TiO_2_)_digesta_)/(Nutrient/TiO_2_)_diet_

### Statistical analysis

Data were checked for normality and then subjected to statistical analysis using 2-way ANOVA of GLM procedure in JMP®13 (SAS Institute Inc., JMP Software, Cary, NC) to assess the main effects of grain source, additive supplementation, and their interaction. Each pen was considered as an experimental unit and the values presented in the tables are means with pooled standard error of mean (SEM). If a significant effect of treatment was detected, differences between treatments or main effects were separated by least square differences test. Significance was considered at *P* < 0.05 and *P* < 0.10 was indicated and discussed as a trend.

## Results

The mortality rate throughout the experimental period was below 3 % and was not affected by dietary treatments (data not shown).

During the starter period, birds offered the wheat-based diet tended (*P* = 0.053) to gain more weight and consumed more feed (*P* < 0.05) compared to those fed the corn-based diet (Table 2). Regardless of feed base grain, both xylanase and stimbiotic supplementation improved FCR over the starter period (*P* < 0.05) compared to non-supplemented birds. As illustrated in Table 3, body weight on day 28 and BWG during the grower period was neither affected by grain source nor enzyme supplementation or their interactions (*P* > 0.05). Over the grower phase, birds fed the corn-based diet presented better FCR (*P* < 0.01) than those fed the wheat-based diet. Stimbiotic supplementation led to significant improvement in grower FCR (*P* < 0.01) compared to non-supplemented birds.

**Table 2:** Growth performance of broiler chickens in response to dietary treatments over the starter period (0–14 d).

Treatment		BW g/b		BWG g/b	FI g/b	FCR g/g
Feed grain	Additive	Day 0	Day 14	0–14 d	0–14 d	0–14 d
Corn	None	34.2	472	438	513	1.171
Corn	Xylanase	34.2	482	448	512	1.142
Corn	Stimbiotic	34.0	485	451	515	1.144
Wheat	None	34.1	494	460	532	1.157
Wheat	Xylanase	34.3	497	463	529	1.143
Wheat	Stimbiotic	34.2	486	452	516	1.140
	SEM	0.078	7.78	7.79	7.17	0.008
*Main effects*						
Feed grain						
Corn		34.2	480	446	513^b^	1.153
Wheat		34.2	492	458	525^a^	1.147
Additive						
None		34.1	483	449	522	1.164^a^
Xylanase		34.2	490	456	520	1.143^b^
Stimbiotic		34.1	485	451	515	1.143^b^
Source of variation *(P-value)*						
Feed grain		0.649	0.052	0.053	0.042	0.384
Additive		0.387	0.668	0.676	0.615	0.012
Grain × Additive		0.176	0.429	0.420	0.351	0.681

Each value for each treatment represents the mean of 8 replicates of 27 birds each.

a-b Means within a column not sharing a superscript differ significantly at the *P* < 0.05 level for the treatment effects and at the P level shown for the main effects.

**Table 3:** Growth performance of broiler chickens in response to dietary treatments over the grower period (14–28 d).

Treatment		BW g/b	BWG g/b	FI g/b	FCR g/g
Feed grain	Additive	Day 28	14–28 d	14–28 d	14–28 d
Corn	None	1766	1294	1743	1.347
Corn	Xylanase	1806	1323	1773	1.340
Corn	Stimbiotic	1788	1303	1723	1.322
Wheat	None	1794	1300	1803	1.387
Wheat	Xylanase	1798	1301	1763	1.355
Wheat	Stimbiotic	1796	1310	1749	1.336
	SEM	16.91	11.34	18.51	0.010
*Main effects*					
Feed grain					
Corn		1786	1306	1747	1.336^b^
Wheat		1795	1303	1771	1.359^a^
Additive					
None		1779	1297	1773	1.367^a^
Xylanase		1801	1312	1767	1.347^a^^b^
Stimbiotic		1791	1306	1736	1.328^b^
Source of variation *(P-value)*					
Feed grain		0.497	0.727	0.098	0.008
Additive		0.431	0.411	0.111	0.002
Grain × Additive		0.577	0.350	0.178	0.361

Each value for each treatment represents the mean of 8 replicates of 27 birds each.

a-b Means within a column not sharing a superscript differ significantly at the *P* < 0.05 level for the treatment effects and at the P level shown for the main effects.

Growth performance over the finisher (28–42 d) and entire production period (0–42 d) is presented in Table 4. The final BW on day 42 was not statistically affected by experimental factors. Interactions between feed grain and the additive supplementation were detected for feed intake over the finisher and overall production period (*P* < 0.05) where stimbiotic supplementation reduced feed intake only in birds fed the wheat-based diet. In addition, there was a feed grain × additive interaction for finisher FCR (*P* < 0.05), in which xylanase or stimbiotic improved the FCR only when supplemented in the wheat-based diet, not in the corn-based diet when compared to non-supplemented birds. Both xylanase and stimbiotic improved the overall FCR compared to non-supplemented birds, regardless of corn- or wheat-based diets. The magnitude of this improvement was greater (∼5 points) in birds fed wheat-barley-based diets compared to that of corn-based diets (∼2 points) which led to a significant interaction between grain source and additive supplementation (*P* < 0.01). However, when the FCR values were corrected to the body weight of control bird, with no additive supplementation, such interaction disappeared, and xylanase and stimbiotic supplementation consistently improved FCR regardless of feed grain source (*P* < 0.01).

**Table 4:** Growth performance of broiler chickens in response to dietary treatments over the finisher (28–42 d) and overall (0–42 d) periods.

Treatment		BW g/b	BWG g/b		FI g/b		FCR g/g			Livability %
Feed grain	Additive	Day 42	28–42 d	0–42 d	28–42 d	0–42 d	28–42 d	0–42 d	BWc^1^	0–42 d
Corn	None	3475	1709	3441	2809^c^	5064^b^	1.644^b^	1.472^b^	1.472	95.8
Corn	Xylanase	3567	1761	3533	2845^b^^c^	5129^b^	1.615^b^	1.452^cd^	1.433	97.1
Corn	Stimbiotic	3561	1774	3527	2863^b^^c^	5101^b^	1.615^b^	1.446^d^	1.429	95.2
Wheat	None	3534	1740	3500	2969^a^	5304^a^	1.706^a^	1.515^a^	1.515	97.0
Wheat	Xylanase	3572	1774	3538	2903^a^^b^	5195^a^^b^	1.637^b^	1.469^bc^	1.461	96.4
Wheat	Stimbiotic	3527	1731	3492	2809^c^	5073^b^	1.622^b^	1.452^bcd^	1.454	94.6
	SEM	32.92	22.03	32.92	33.20	50.65	0.010	0.006	0.009	1.85
*Main effects*										
Feed grain										
Corn		3534	1748	3500	2838	5098	1.624	1.456	1.444^b^	96.1
Wheat		3544	1748	3510	2893	5191	1.655	1.478	1.476^a^	96.0
Additive										
None		3504	1724	3470	2888	5184	1.675	1.493	1.493^a^	96.4
Xylanase		3569	1767	3535	2874	5162	1.625	1.460	1.446^b^	96.7
Stimbiotic		3544	1752	3510	2835	5087	1.618	1.449	1.441^b^	94.9
Source of variation *(P-value)*										
Feed grain		0.711	0.975	0.712	0.047	0.029	0.004	< 0.001	< 0.001	0.987
Additive		0.148	0.151	0.148	0.269	0.147	< 0.001	< 0.001	< 0.001	0.582
Grain × Additive		0.366	0.226	0.364	0.009	0.035	0.035	0.009	0.570	0.851

Each value for each treatment represents the mean of 8 replicates of 27 birds each.

a-d Means within a column not sharing a superscript differ significantly at the *P* < 0.05 level for the treatment effects and at the P level shown for the main effects.

1 FCR values corrected to the BW of control birds in treatment 1 and 4 by considering 50 g difference in BW equivalent to 1.0 (0.01) point of FCR

Duodenal, jejunal and ileal digesta viscosity measured at day 27 of experiment are shown in Table 5. As a main effect, birds fed wheat-barley based diets showed higher duodenal and jejunal viscosity than birds fed corn-based diets (*P* < 0.01). Also, additive supplementation tended (*P* = 0.094) to reduce duodenal viscosity. There was an interaction between feed grain and additive supplementation on ileal digesta viscosity (*P* < 0.01), where either xylanase or stimbiotic reduced the viscosity only in birds fed the wheat-barley-based diet compared to those offered the corn-based diet. A similar tendency (*P* = 0.097) was observed with the jejunal digesta viscosity reduction by additive supplementation in the wheat-barley-fed birds compared to the corn-fed birds.

**Table 5:** Small intestinal digesta viscosity and ileal digestibility coefficient of nutrients at 27 days of age; and serum fluorescein isothiocyanate-dextran (FITC-d) concentration at 21 days of age in response to dietary treatments.

Treatment		Viscosity (m·Pas) at d 27			Ileal digestibility coefficient at d 27			Serum FITC-d at d 21, µg/mL
Feed grain	Additive	Duodenum	Jejunum	Ileum	Protein	Energy	Starch	
Corn	None	2.021	1.803	2.320^c^	0.836^a^	0.758	0.975^a^	0.274
Corn	Xylanase	2.088	1.854	2.330^c^	0.840^a^	0.763	0.976^a^	0.276
Corn	Stimbiotic	1.811	1.784	2.314^c^	0.844^a^	0.773	0.985^a^	0.275
Wheat	None	2.460	2.631	3.219^a^	0.785^c^	0.702	0.952^b^	0.258
Wheat	Xylanase	2.208	2.265	2.636^b^	0.807^b^	0.714	0.977^a^	0.259
Wheat	Stimbiotic	2.126	2.258	2.688^b^	0.813^b^	0.720	0.981^a^	0.254
	SEM	0.123	0.101	0.068	0.004	0.005	0.003	0.003
*Main effects*								
Feed grain								
Corn		1.973^b^	1.813^b^	2.321	0.840	0.765^a^	0.979	0.275^a^
Wheat		2.264^a^	2.384^a^	2.847	0.802	0.712^b^	0.970	0.257^b^
Additive								
None		2.241	2.217	2.769	0.811	0.731^b^	0.964	0.266
Xylanase		2.147	2.059	2.483	0.824	0.739^a^^b^	0.976	0.267
Stimbiotic		1.968	2.021	2.501	0.829	0.747^a^	0.982	0.265
Source of variation *(P-value)*								
Feed grain		0.006	< 0.001	< 0.001	< 0.001	< 0.001	0.004	< 0.001
Additive		0.094	0.135	0.001	0.001	0.024	< 0.001	0.748
Grain × Additive		0.436	0.097	0.001	0.016	0.837	0.006	0.819

Each value for each treatment represents the mean of 8 replicates with 3 birds per pen selected for the above analysis.

a-d Means within a column not sharing a superscript differ significantly at the *P* < 0.05 level for the treatment effects and at the P level shown for the main effects.

A significant (*P* < 0.05) interaction between additive supplementation and grain source resulted in improved protein digestibility by an average of 3.2 % in response to xylanase and stimbiotic supplementation only in birds fed wheat-barley based diets. Birds fed corn-based diets had higher ileal energy digestibility than those fed wheat-barley based diets (*P* < 0.05). As the main effect and irrespective of dietary grain source, stimbiotic supplementation improved ileal energy digestibility by an average of 2.2 % (*P* < 0.05). Both xylanase and stimbiotic improved starch digestibility in wheat-barley based diets leading to an interaction between the additive supplementation and grain source (*P* < 0.01).

Feeding corn-based diets increased the passage of FITC-d from the intestine into the blood by around 7.0 %, indicating higher intestinal permeability compared to wheat-barley diets (*P* < 0.01), but there was no significant effect of xylanase or stimbiotic on serum FITC-d concentration (*P* > 0.05).

## Discussion

In general, supplementing xylanase or stimbiotic positively influenced feed efficiency and energy utilisation in broiler chickens, irrespective of diet base grains. However, feed grains appeared to dictate the mechanism of action of the additives and affected birds’ performance differently. With the corn-based diet, the FCR improvement was closely associated with increased weight gain whereas with the wheat-based diet it was with reduced feed intake. A possible assumption is that supplementing xylanase and stimbiotic to the wheat-based diet may have promoted fibre fermentation and production of short-chain fatty acids as an additional source of energy, which could have contributed to improved feed efficiency along with lower feed consumption. Similarly, Morgan et al. (2022) observed improved feed efficiency associated with decreased feed intake upon xylanase supplementation in broilers fed diets based on wheat. In the present study, both xylanase and stimbiotic supplementations markedly reduced the digesta viscosity throughout the small intestine only in wheat-barley fed birds. This suggests successful hydrolysis of wheat arabinoxylans upon supplementation of xylanase and stimbiotic. Furthermore, the improved FCR may be associated with reduced digesta viscosity, indicating depolymerization and the formation of more readily fermentable, medium-chain-length soluble arabinoxylans. As expected, corn-based diets with lower soluble NSP contents did not create a viscous gut environment even in the absence of xylanase or stimbiotic. Although not statistically significant, the corn-based diets supplemented with xylanase or stimbiotic led to numerically higher energy and protein digestibility at the ileal level than non-supplemented diets. It is assumed that the positive impacts of xylanase and stimbiotic with corn-based diets resulted from the release of entrapped nutrients followed by partial solubilisation of insoluble arabinoxylans (Bautil et al., 2021).

The extent of improvements made by feed enzymes is dictated by the affinity of enzymes and the presence and quantity of target substrates available in feed (Kim et al., 2022). Most microbial xylanases act on both soluble and insoluble xylans but are preferentially more active against soluble and unsubstituted fractions (Beaugrand et al., 2004; Moreira & Filho, 2016). Wheat is relatively high in concentrations of both soluble and insoluble arabinoxylans that can be targeted by microbial xylanases to a greater extent when compared with other common ingredients. Corn also contains appreciable amounts of arabinoxylans as a major type of NSP, but they are mostly present in insoluble form and with a degree of substitution that makes them less susceptible to xylanase attack (Bach Knudsen, 2014). Therefore, a greater magnitude of xylanase-mediated improvement in growth performance and nutrient digestibility could be achieved when supplemented to wheat-containing diets. However, it should be noted that the current study used up to 25 % of barley in the wheat-based diets, which may have partially masked the positive impacts of the additives on wheat arabinoxylans. Barely contain high-molecular weight, soluble β-glucans (Bach Knudsen, 2014), which also can hinder efficient nutrient digestion in poultry; however, both additives used in the present study were not counteractive to β-glucans.

Impaired protein utilisation due to soluble NSP present in viscous diets could lead to protein fermentation in the hindgut, resulting in undesirable harmful metabolites such as ammonia and amines (Apajalahti & Vienola, 2016; Nakata et al., 2017). Moreover, wheat and barley contain higher crude protein contents compared to corn; therefore, there is a greater chance that protein escape small intestinal digestion, leading to the undesired protein fermentation in the hindgut. Hence, the undigested protein may fuel putrefactive bacteria to flourish in the lower gut, giving less room for beneficial bacteria species to establish (Qaisrani et al., 2015). Supplemental xylanase can improve protein digestibility by breaking down grain endosperm cell walls and alleviating the cage effect of insoluble NSP. Furthermore, the hydrolysis of NSP by xylanase releases more readily fermentable fibre that favourably increases the ratio of fermentable carbohydrates to protein entering the caeca for bacterial fermentation. Our results showed that in the absence of additives, birds offered the wheat-based diet showed markedly lower protein digestibility at the ileal level relative to those fed the corn-based diet. This was successfully countered by supplemental xylanase or stimbiotic, which, in turn, positively affected overall FCR in birds fed the wheat-based diet. Future trials should incorporate additional gastrointestinal health metrics and evaluate responses under disease-challenged conditions to better understand the mechanisms driving nutrient digestibility improvements associated with xylanase and/or stimbiotic supplementation.

The advantage of combining xylanase and fermentable xylo-oligosaccharides in the stimbiotic product appeared to be marginal compared to single xylanase supplementation on most of the measurements explored in the present study. Supplementing fermentable oligosaccharides is expected to boost the early development of the intestinal microbiome, selectively stimulating fibre-degrading bacteria even at very low doses (Bautil et al., 2020; Chen et al., 2023; Ribeiro et al., 2018). However, growth performance responses of birds to stimbiotics have not been consistent. González-Ortiz et al. (2021) found that supplementing a combination of xylanase and xylo-oligosaccharides to corn-wheat-soybean meal-based diets could significantly improve growth performance compared to non-supplemented diets, with more pronounced improvement seen when the feed was deficient in energy and amino acids. On the contrary, Singh et al. (2021) reported that corn-based diets supplemented with stimbiotic promoted caecal short-chain fatty acids (SCFAs) production, but this only translated into a numerical improvement in the weight gain. The latter is in accordance with our findings where stimbiotic led to a numerical improvement in overall FCR compared to the single xylanase supplementation across the diet types. When supplemented in a wheat-barley-based diet, xylanase alone likely releases sufficient readily fermentable, lower-molecular-weight xylo-oligosaccharides following NSP breakdown. As a result, the additional xylo-oligosaccharides provided by the stimbiotic may offer limited benefits in healthy birds compared to challenge conditions (Lee et al., 2022). In contrast, corn-based diets are relatively low in soluble NSP, limiting their interaction with supplemental xylanase. Šimić et al. (2023) also highlighted that the presence of soluble arabinoxylans in the diet is crucial for expanding cecal fibre fermentation capacity when supplemental xylanase, xylo-oligosaccharides, or stimbiotics are used in corn-based diets. Further research is needed to optimize dietary fibre sources and the use of supplemental stimbiotics to achieve consistent improvements in bird growth performance.

Intestinal permeability (IP), arising from disruptions in the physical barrier, refers to the permeability of the intestinal epithelium (Gilani et al., 2021). It is often characterized by electrophysiologists using Ussing Chambers in *in vitro* tissue studies, making it a measurable feature of intestinal barrier integrity (Clarke, 2009; Hering et al., 2012). Among the assays available, fluorescein isothiocyanate dextran (FITC-d; 3,000 to 5,000 Da), which traverses paracellular pathways, is the most widely used method for assessing IP in chickens. Elevated blood FITC-d levels can indicate compromised IP, although some permeability is normal in healthy animals, as the intestinal lining is not a completely sealed barrier (Tooley et al., 2009). Numerous studies have documented compromised IP under challenge conditions, including fasting (Maguey-Gonzalez et al., 2018), heat stress (Shakeri et al., 2018), coccidiosis (Chadwick et al., 2020) and necrotic enteritis challenge (Oxford and Selvaraj, 2019). However, research on changes in IP in response to diet composition or feed additives under non-challenge conditions remains limited. Baxter et al. (2019) reported that broiler chickens fed a rye-based diet exhibited increased serum levels of FITC-d compared to those fed a corn-based diet, indicating higher IP. Interestingly, in this study, birds fed corn-based diets exhibited higher IP compared to those fed wheat-barley-based diets, with no significant main effect or interaction observed for xylanase and stimbiotic supplementation. Both wheat and barley are considered as viscous grains and although the soluble NSPs in wheat and barley can increase digesta viscosity (Meng et al., 2005), which was also observed in this study, they may also stimulate mucus secretion, which can protect the gut lining. Hydrolysed or partially hydrolysed NSPs in wheat and barley can ferment in the hindgut, producing SCFAs such as butyrate (Józefiak et al., 2004). Higher digesta concentrations of acetic and butyric acids has been reported in birds fed wheat-based diets compared to birds fed corn-based diets (Kiarie et al., 2014). Butyrate is known to strengthen tight junction proteins, including claudins and occludins, in intestinal epithelial cells, thereby enhancing gut barrier integrity and reducing permeability (Wang et al., 2012). Additionally, β-glucans from barley have immune-modulating properties, which can reduce gut inflammation (Jacob and Pescatore, 2017). Since inflammation is a key driver of increased IP, a less inflamed gut is inherently less permeable.

## Conclusion

This study confirmed the benefits of xylanase and stimbiotic supplementation in broiler diets through consistent improvements in feed efficiency and energy utilization across both corn- and wheat-barley-based diets. The mechanisms of action and performance responses were grain specific. In wheat-barley diets, improvements were associated with reduced digesta viscosity, lower feed intake, and potentially enhanced fiber fermentation, leading to better protein and starch digestibility. Conversely, the benefits observed in corn-based diets were not linked to reductions in intestinal viscosity or enhanced nutrient digestibility but may be attributed to the enzymatic degradation of arabinoxylans, releasing arabinoxylo-oligosaccharides that act as prebiotics to support beneficial gut microbiota. Although both xylanase and stimbiotic supplementation reduced digesta viscosity in wheat-barley diets, the added value of stimbiotics over xylanase alone appeared limited under the unchallenged conditions of this study, indicating that xylanase may suffice to counteract the anti-nutritive effects of non-starch polysaccharides and may generate stimbiotic XOS *in situ.* These results also suggest that intestinal permeability, a key indicator of gut barrier function, can be modulated by the type of cereal grain in the diet. Soluble NSPs in wheat and barley increase digesta viscosity but may also enhance the production of beneficial short-chain fatty acids, such as butyrate, which enhance gut barrier function. Furthermore, the anti-inflammatory properties of β-glucans from barley may contribute to reduced intestinal permeability.

## Declaration of competing interest

We declare that we have no financial and personal relationships with other people or organizations that can inappropriately influence our work, and there is no professional or other personal interest of any nature or kind in any product, service and/or company that could be construed as influencing the content of this paper.

## References

[bib0001] AOAC International (2012). Official Methods of Analysis.

[bib0002] Amerah A.M., Romero L.F., Awati A., Ravindran V. (2017). Effect of exogenous xylanase, amylase, and protease as single or combined activities on nutrient digestibility and growth performance of broilers fed corn/soy diets. Poult. Sci..

[bib0003] Annison G. (1991). Relationship between the levels of soluble nonstarch polysaccharides and the apparent metabolizable energy of wheats assayed in broiler chickens. J. Agric. Food Chem..

[bib0004] Apajalahti J., Vienola K. (2016). Interaction between chicken intestinal microbiota and protein digestion. Anim. Feed Sci. Technol..

[bib0005] Bach Knudsen K.E. (2014). Fiber and nonstarch polysaccharide content and variation in common crops used in broiler diets. Poult. Sci..

[bib0006] Barekatain R., Nattrass G., Tilbrook A.J., Chousalkar K., Gilani S. (2019). Reduced protein diet and amino acid concentration alter intestinal barrier function and performance of broiler chickens with or without synthetic glucocorticoid. Poult. Sci..

[bib0007] Bautil A., Buyse J., Goos P., Bedford M.R., Courtin C.M. (2021). Feed endoxylanase type and dose affect arabinoxylan hydrolysis and fermentation in ageing broilers. Anim. Nutr..

[bib0008] Bautil A., Verspreet J., Buyse J., Goos P., Bedford M., Courtin C. (2020). Arabinoxylan-oligosaccharides kick-start arabinoxylan digestion in the aging broiler. Poult. Sci..

[bib0009] Baxter M.F., Dridi S., Koltes D.A., Latorre J.D., Bottje W.G., Greene E.S., Bickler S.W., Kim J.H., Merino-Guzman R., Hernandez-Velasco X., Anthony N.B. (2019). Evaluation of intestinal permeability and liver bacterial translocation in two modern broilers and their jungle fowl ancestor. Front. Genet..

[bib0010] Beaugrand J., Chambat G., Wong V.W., Goubet F., Rémond C., Paës G., Benamrouche S., Debeire P., O’Donohue M., Chabbert B. (2004). Impact and efficiency of GH10 and GH11 thermostable endoxylanases on wheat bran and alkali-extractable arabinoxylans. Carbohydr. Res..

[bib0011] Bedford M., Cowieson A.J. (2012). Exogenous enzymes and their effects on intestinal microbiology. Anim. Feed Sci. Technol..

[bib0012] Bedford, M.R., C.L. Walk, and M. Hruby. 2022. Evolving enzyme Applications. Pages 286-290 in Enzymes in farm animal nutrition. Ed. M.R. Bedford, G.G. Partridge, M. Hruby and C. L. Walk, Oxfordshire, UK.

[bib0013] Chadwick E., Rahimi S., Grimes J., Pitts J., Beckstead R. (2020). Sodium bisulfate feed additive aids broilers in growth and intestinal health during a coccidiosis challenge. Poult. Sci..

[bib0014] Chen W., Yin C., Li J., Sun W., Li Y., Wang C., Pi Y., Cordero G., Li X., Jiang X. (2023). Stimbiotics supplementation promotes growth performance by improving plasma immunoglobulin and IGF-1 levels and regulating gut microbiota composition in weaned piglets. Biology.

[bib0015] Choct M. (2015). Feed non-starch polysaccharides for monogastric animals: classification and function. Anim. Prod. Sci..

[bib0016] Clarke L.L. (2009). A guide to Ussing chamber studies of mouse intestine. Am. J. Physiol. Gastrointest. Liver Physiol..

[bib0017] Davies C., González-Ortiz G., Rinttilä T., Apajalahti J., Alyassin M., Bedford M.R. (2024). Stimbiotic supplementation and xylose-rich carbohydrates modulate broiler’s capacity to ferment fibre. Front. Microbiol..

[bib0018] Gilani S., Chrystal P.V., Barekatain R. (2021). Current experimental models, assessment and dietary modulations of intestinal permeability in broiler chickens. Anim. Nutr..

[bib0020] Gonzalez-Ortiz G., Sola-Oriol D., Martinez-Mora M., Perez J.F., Bedford M.R. (2017). Response of broiler chickens fed wheat-based diets to xylanase supplementation. Poult. Sci..

[bib0021] González-Ortiz, G., Bedford, M.R., Bach Knudsen, K.E., Courtin, C.M., and Classen, H.L. (eds.). 2019. *The value of fibre: Engaging the second brain for animal nutrition*. Wageningen Academic Publishers, Wageningen, the Netherlands.

[bib0022] Hering N.A., Fromm M., Schulzke J.D. (2012). Determinants of colonic barrier function in inflammatory bowel disease and potential therapeutics. J. Physiol..

[bib0023] Jacob J., Pescatore A. (2017). Glucans and the poultry immune system. Am. J. Immunol..

[bib0024] Józefiak D., Rutkowski A., Fratczak M., Boros D. (2004). The effect of dietary fiberfractions from different cereals and microbial enzyme supplementation on performance, ileal viscosity and short-chain fatty acid concentrations in the caeca of broiler chickens. J. Anim. Feed Sci..

[bib0025] Kiarie E., Romero L.F., Ravindran V. (2014). Growth performance, nutrient utilization, and digesta characteristics in broiler chickens fed corn or wheat diets without or with supplemental xylanase. Poult. Sci..

[bib0026] Kim E., Moss A.F., Morgan N.K., Gharib-Naseri K., Ader P., Choct M. (2022). Ileal profile of non-starch polysaccharides and oligosaccharides in response to exogenous enzymes in broiler chickens offered wheat-or maize-based diets under subclinical necrotic enteritis challenge. Anim. Nutr..

[bib0027] Lee J.H., Lee B., Rousseau X., Gomes G.A., Oh H.J., Kim Y.J., Chang S.Y., An J.W., Go Y.B., Song D.C., Cho H.A. (2022). Stimbiotic supplementation modulated intestinal inflammatory response and improved broilers performance in an experimentally-induced necrotic enteritis infection model. J. Anim. Sci. Biotechnol..

[bib0028] Meng X., Slominski B.A., Nyachoti C.M., Campbell L.D., Guenter W. (2005). Degradation of cell wall polysaccharides by combinations of carbohydrase enzymes and their effect on nutrient utilization and broiler chicken performance. Poult. Sci..

[bib0029] Morgan N.K., Bhuiyan M., Wallace A., Hopcroft R. (2022). Comparing a single dose of xylanase to a double dose or cocktail of non-starch polysaccharide-degrading enzymes in broiler chicken diets. J. Appl. Anim. Nutr..

[bib0030] Morgan N.K., Wallace A., Bedford M.R., Hawking K.L., Rodrigues I., Hilliar M., Choct M. (2020). In vitro versus in situ evaluation of xylan hydrolysis into xylo-oligosaccharides in broiler chicken gastrointestinal tract. Carbohydr. Polym..

[bib0031] Moreira L., Filho E. (2016). Insights into the mechanism of enzymatic hydrolysis of xylan. Appl. Microbiol. Biotechnol..

[bib0032] Maguey-Gonzalez J.A., Michel M.A., Baxter M.F., Tellez Jr G., Moore Jr P.A., Solis-Cruz B., Hernández-Patlan D., Merino-Guzman R., Hernandez-Velasco X., Latorre J.D., Hargis B.M. (2018). Effect of humic acids on intestinal viscosity, leaky gut, and ammonia excretion in a 24-hour feed restriction model to induce intestinal permeability in broiler chickens. Anim. Sci. J..

[bib0033] Nakata T., Kyoui D., Takahashi H., Kimura B., Kuda T. (2017). Inhibitory effects of soybean oligosaccharides and water-soluble soybean fiberon formation of putrefactive compounds from soy protein by gut microbiota. Int. J. Biol. Macromol..

[bib0034] Nguyen H.T., Bedford M.R., Wu S., Morgan N.K. (2022). Dietary soluble non-starch polysaccharide level influences performance, nutrient utilization, and disappearance of non-starch polysaccharides in broiler chickens. Animals.

[bib0035] Nguyen H., Bedford M., Wu S-B., Morgan N.K. (2021). Soluble non-starch polysaccharide modulates broiler gastrointestinal tract environment. Poult. Sci..

[bib0036] Nieto-Domínguez M., de Eugenio L.I., York-Durán M.J., Rodríguez-Colinas B., Plou F.J., Chenoll E., Pardo E., Codoñer F., Martínez M.J. (2017). Prebiotic effect of xylooligosaccharides produced from birchwood xylan by a novel fungal GH11 xylanase. Food Chem..

[bib0037] Oxford J.H., Selvaraj R.K. (2019). Effects of glutamine supplementation on broiler performance and intestinal immune parameters during an experimental coccidiosis infection. J. Appl. Poult. Res..

[bib0038] Qaisrani S., Van Krimpen M., Kwakkel R., Verstegen M., Hendriks W. (2015). Dietary factors affecting hindgut protein fermentation in broilers: a review. World. Poult. Sci. J..

[bib0039] Ribeiro T., Cardoso V., Ferreira L., Lordelo M., Coelho E., Moreira A., Domingues M., Coimbra M., Bedford M., Fontes C. (2018). Xylo-oligosaccharides display a prebiotic activity when used to supplement wheat or corn-based diets for broilers. Poult. Sci..

[bib0040] Shakeri M., Cottrell J.J., Wilkinson S., Ringuet M., Furness J.B., Dunshea F.R. (2018). Betaine and antioxidants improve growth performance, breast muscle development, and ameliorate thermoregulatory responses to cyclic heat exposure in broiler chickens. Animals.

[bib0041] Short F., Gorton P., Wiseman J., Boorman K. (1996). Determination of titanium dioxide added as an inert marker in chicken digestibility studies. Anim. Feed Sci. Technol..

[bib0042] Šimić A., González-Ortiz G., Mansbridge S.C., Rose S.P., Bedford M.R., Yovchev D., Pirgozliev V.R. (2023). Broiler chicken response to xylanase and fermentable xylooligosaccharide supplementation. Poult. Sci..

[bib0043] Singh A.K., Mishra B., Bedford M.R., Jha R. (2021). Effects of supplemental xylanase and xylooligosaccharides on production performance and gut health variables of broiler chickens. J. Anim. Sci. Biotechnol..

[bib0044] Tooley K.L., Howarth G.S., Butler R.N. (2009). Mucositis and non-invasive markers of small intestinal function. Cancer Biol. Ther..

[bib0045] Wang H.B., Wang P.Y., Wang X., Wan Y.L., Liu Y.C. (2012). Butyrate enhances intestinal epithelial barrier function via up-regulation of tight junction protein Claudin-1 transcription. Dig. Dis. Sci..

